# Draft Genome Sequences of *Salmonella enterica* subsp. *enterica* Serovar Berta ATCC 8392 and a Nalidixic Acid-Resistant Isolate of This Strain

**DOI:** 10.1128/genomeA.00186-16

**Published:** 2016-04-21

**Authors:** Ashley Cooper, Adam G. Koziol, Catherine D. Carrillo, Dominic Lambert

**Affiliations:** Canadian Food Inspection Agency, Government of Canada, Ottawa, Canada

## Abstract

*Salmonella enterica* subspecies *enterica* serovar Berta has been isolated in multiple animal species and has been implicated in human disease. Here, we report a 4.7-Mbp draft genome sequence of *S. enterica* serovar Berta (ATCC strain 8392) and a nalidixic acid-resistant isolate derived from this strain.

## GENOME ANNOUNCEMENT

*Salmonella enterica* serovar Berta was first isolated from pig mesenteric glands in Uruguay by Hormaeche and Salsamendi in 1936 (1). Years later the first human outbreak was reported following food poisoning from *S*. *enterica* serovar Berta contaminated pork sausage (2). This organism has since been isolated from cheese, poultry, cattle, humans, and some cold-blooded animals (3–6). More recent illnesses associated with *S*. *enterica* serovar Berta have occurred in Italy following consumption of dairy-based desserts and eggs (7) as well as a rare case of *S*. *enterica* serovar Berta meningitis in a neonate (8). *S*. *enterica* serovar Berta is characterized by antigenic group O9 (formerly D_1_) and contains somatic O antigens 1, 9, 12, and flagellar H antigens [f], g, [t] (phase 1) (9). Additionally, *S*. *enterica* serovar Berta is atypical in that it lacks the capacity to produce hydrogen sulfide (H_2_S), a reaction usually utilized to identify salmonellae (10).

*S*. *enterica* serovar Berta type strain ATCC 8392 and a nalidixic acid-resistant (NALR) isolate derived from this strain were analyzed in this study. Genomic DNA was isolated from overnight cultures grown on brain heart infusion (BHI) agar using the Promega, Maxwell 16 cell DNA purification kit (Promega, Madison, WI). Sequencing libraries were constructed using the Nextera XT DNA sample preparation kit (Illumina, Inc., San Diego, CA) and paired-end sequencing was performed on the Illumina MiSeq platform (Illumina, Inc.), using a 600 cycle MiSeq reagent kit (v3). Sequencing errors in reads were corrected using Quake version 0.3 with a k-mer size of 15 (11) and assembled *de novo* using SPAdes v3.1.1 (12). Contigs shorter than 1000 bp were excluded from the analysis.

Draft genomes of the isolates of *S*. *enterica* serovar Berta ATCC 8392 characterized in this study were very similar. The genome coverage, combined length of genome size, and G+C content were 32, 4.7-Mbp, and 52.3%, respectively, for both. For *S*. *enterica* serovar Berta ATCC 8392 and *S*. *enterica* serovar Berta ATCC 8392 NALR, the number of paired-end reads were 1,095,369, and 1,336,793, and the number of contigs larger than 200 bp were 67 and 44, respectively. Gene predictions and annotations were performed with the National Center for Biotechnology Information (NCBI) Prokaryotic Genome Annotation Pipeline (PGAP) (13), which predicted 4,437 and 4,435 coding sequences (CDS) for *S*. *enterica* serovar Berta and *S*. *enterica* serovar Berta NALR, respectively.

There were three single nucleotide differences in the *S*. *enterica* serovar Berta NALR relative to the parent strain, as determined using kSNP (version 3.0 with a kmer value of 51) (14). One of these was a single point mutation in the gene encoding gyrase (*gyrA*), resulting in an 87-GAC (Asp) → AAC (Asn) mutation. Point mutations in *gyrA* are commonly associated with NALR in salmonellae (15, 16). In both genomes, a mutation resulting in a 455-CAA (Gln) → UAA (Stop) was observed in the phs operon in the *phsA* thiosulfate reductase gene, which plays a role in H_2_S production (17). Similarly, mutations in *phsA* resulting in premature stop codons have been associated with non-H_2_S-producing *S. enterica* serovars Typhimurium and Infantis isolates in Japan (18).

### Nucleotide sequence accession numbers.

This whole-genome shotgun project has been deposited at DDBJ/EMBL/GenBank under the accession numbers JZUW00000000 and JZUV00000000 for *S*. *enterica* serovar Berta and *S*. *enterica* serovar Berta NALR, respectively. The versions described in this paper are the first versions.
